# The traditional practice of canine bud removal in the offspring of Ethiopian immigrants

**DOI:** 10.1186/1472-6831-13-34

**Published:** 2013-07-19

**Authors:** Esti Davidovich, Eli Kooby, Joseph Shapira, Diana Ram

**Affiliations:** 1 Department of Pediatric Dentistry, The Hebrew University-Hadassah School of Dental Medicine, Jerusalem, Israel; 2 Ministry of Health, Head, Department of Oral Health, Ministry of Health, Tel Aviv, Israel

**Keywords:** Canine bud removal, Enamel defects, Infant oral mutilation, Immigrants, Pediatric dental care

## Abstract

**Background:**

The custom of canine bud removal has detrimental consequences on children’s general health and dental care. The objective of this study was to assess whether the prevalence of missing primary canines and dental defects in offspring of emigrants from Ethiopia is greater than in offspring of native Israeli parents of similar socioeconomic class.

**Methods:**

477 children of Ethiopian descent and 317 offspring of native Israeli parents, from 21 nursery schools and kindergartens, underwent dental examinations aimed to determine the presence or absence of primary canines and of developmental enamel defects on adjacent teeth to the primary canines. For purposes of analysis, children were classified into two age groups: younger (ages 18–48 months) and older (ages 49–82 months).

**Results:**

Canines were present in more Israeli than Ethiopian younger children, 87.5% vs. 42.3%, p=0.0001; and in more Israeli than Ethiopian older children, 92.6% vs. 40.4%, p=0.0001. More dental defects were detected in Ethiopian than in Israeli younger children, 32% vs. 3.9%, p=0.0001; and in more Ethiopian than Israeli older children, 31.2% vs. 5.8%, p=0.0001.

**Conclusions:**

The prevalence of missing primary canines and dental defects was greater among offspring of parents who had emigrated from Ethiopia 15–20 years earlier than among offspring of native Israeli parents living in the same low socioeconomic neighborhoods.

## Background

As more people from developing countries in Africa migrate to industrialized countries, more health professionals need to provide care to people with customs and practices of which they have little knowledge [1]. More than 40,000 individuals have immigrated to Israel from Ethiopia in the last 30 years. About 10,000 children have been born to Ethiopian parents in Israel.

Infant oral mutilation (IOM) differs from more recognized practices of culturally based dental modification in several important aspects: it is performed for perceived medical benefit; it is performed in infants who are incapable of consenting to the practice; and it can have very serious health consequences [2]. The removal of incipient canine teeth in babies is practiced in many parts of Africa [3-11], with prevalence rates documented at 22% in Sudan [3], 17.2% in Uganda [4], 37.4% in Tanzania [5], and 30% in Ethiopia [6]. Although apparently rare in developed countries, canine bud removal has been reported in countries that absorb immigrants from Africa, such as the United States [2] and England [7]. In a study conducted two decades ago in Israel, canine bud extraction was evident in 59% of children examined in an Ethiopian community [8]. The procedure is carried out by traditional healers, respected older women, family members or others, using fingernails or sharp instruments such as hot needles or knives. The extraction leads to heavy bleeding, infection, inflammation, and acute suffering, since it is typically performed without anesthesia and generates dental defects in the successor permanent teeth, or in the primary canine and the adjacent primary teeth if not all the bud is extracted [1]. Some children die from uncontrolled bleeding or from sepsis subsequent to contamination of the wound due to the use of unsterilized instruments. HIV transmission is also a risk. Moreover, affected children do not receive proper treatment for their illness [6].

The procedure of bud removal can lead to loss of the canine (in case of complete bud removal) or to hypoplasia or opacity of the tooth and the adjacent teeth, depending on the degree of local trauma inflicted on the bud that could not be removed during the procedure. The late sequel of this practice includes: retention of primary lateral incisor with distal eruption of the succedaneous teeth, hypoplasia of the adjacent primary and permanent teeth, development of odontoma-like structures, midline and occlusal discrepancies, failure of development of succedaneous teeth, displacement and impaction of the permanent teeth, ectopic eruption, development of peg-shaped and invaginated teeth, transpositions, and early eruption of permanent teeth where the primary buds where removed [9].

The aim of the current study was to compare the prevalence of missing primary canines and dental defects (enamel hypoplasia and opacities) in offspring of immigrants from Ethiopia to that of offspring of native Israelis, residing in the same neighborhoods, and attending the same nursery schools and kindergartens.

## Methods

In this cross sectional study we compared the prevalence of missing primary canines and dental defects in Israeli offspring of immigrants from Ethiopia to that of offspring of native Israelis living in the same neighborhoods. Twenty-one nursery schools and kindergartens located in low socioeconomic neighborhoods, in which at least 25% of the children were born to offspring of immigrants from Ethiopia, were included in the survey. Children were examined following approval by the Institutional Review Board for research on human subjects of the Israel Ministry of Health, and following attainment of informed consent from all parents.

The survey was part of a program of the Israel Ministry of Health, conducted during the years 2005–2008, to assess the dental needs of children of Ethiopian immigrants. Children were examined at the participating nursery schools and kindergartens, while sitting on their parents’ laps, under room light, and with a dental mirror and probe, according to World Health Organization (WHO) guidelines [12]. All children were examined by a single examiner, a specialist in pediatric dentistry (ED), one dental assistant, and a secretary who recorded the findings.

The data collected included demographic information (age, gender and ethnic origin) and findings of a clinical examination, including the location, if present, of intact primary canines and dental defects. The latter included enamel and dentin hypoplasia, hypocalcification and opacities. In addition, enamel defects of the adjacent teeth to the primary canines were also recorded, according to the enamel defects index (DDE) [13]. Canines were considered intact when no clinical defect was visible and the teeth looked normal. Canines were considered to have enamel defects when enamel hypoplasia (appearing as pits, grooves, or generalized lack of surface enamel) or opacities (intact surfaces, white, yellow, or brown in color, and round to oval in shape) presented. A missing canine was considered when no canine was present. Enamel defects of the adjacent teeth were considered when the lateral or first primary tooth (adjacent to the canine) presented any of the above mentioned defects.

### Statistical analysis

For purposes of analysis, children were classified into two age groups: younger (ages 18–48 months) and older (ages 49–82 months). Frequencies and percentages were calculated for the categorical variables. Frequencies of the categorical variables between pairs of subgroups were compared by the Chi square test (a parametric test) or by the “Fisher-Irwin exact test (a non-parametric test for small samples). The level of significance for all tests was p≤0.05.

## Results

Of the 860 children who attended the participating nurseries and kindergartens, 794 (92%) were examined: 477 of Ethiopian descent and 317 offspring of native Israeli parents; 350 were younger and 444 older children, according to the categories described above. Canines were present in more children of native Israelis than in children of Ethiopians, in both the younger group, 87.5% vs. 42.3%, p=0.0001 and the older group, 92.6% vs. 40.4%, p=0.0001 (Table 1). Among children with missing canines, both lower canines were missing in 84.4%, all four canines in 4%, and one lower canine (either right or left) in 11.6%.

**Table 1:** **Children**’**s canine status according to parents**’ **cultural background**

**Canine status**	**Younger children (%)**		**Older children number (%)**	
	**Number (%)**			
	**Ethiopian**	**Native Israeli**	**Ethiopian**	**Native Israeli**
**Intact**	**94** (**42**.**3**)	**112** (**87**.**5**)	**103** (**40**.**4**)	**1**75 (92.6)
**With enamel defects**	69 (31.1)	4 (3.1)	76 (29.8)	7 (3.7)
**Missing**	59 (26.6)	12 (9.4)	76 (29.8)	7 (3.7)
**Total**	222 (100)	128 (100)	255 (100)	189 (100)
**P value**	**0**.**0001**		**0**.**0001**	

Among the younger children, 71 (32%) of offspring of Ethiopian parents and 5 (3.9%) of native Israelis presented with dental defects in the adjacent teeth of the missing canines (lateral or first primary molar). In the older group, the respective rates were 82 children (31.2%) of the Ethiopian group and 11 (5.8%) of the native Israeli group.

## Discussion

Among children living in the same neighborhoods and attending the same nursery schools and kindergartens, the prevalence of missing primary canines and dental defects were significantly greater among offspring of immigrants from Ethiopia than among offspring of native Israel parents. Missing canines in the primary dentition is generally a rare finding [14]. However, it is common among people from Eastern Africa due to the practice of bud extraction [3-11].

In the current study, nearly 60% of Ethiopian children, both younger and older, presented with either missing canines or with dental defects of the canines. This contrasts with 12.5% of younger, and 7.4% of older offspring of native Israelis. These findings suggest that canine bud removal is still practiced in children whose parents emigrated from Ethiopia 15–20 years earlier, and reflect a negative reaction to, or ignorance of, western-style, allopathic medicine. The missing canines and the enamel defects of the canines apparently resulted from failure of the procedure to completely remove the bud. In many cases part of the bud remained and erupted as a defective tooth.

The occurrence, though infrequent, of missing canines in the native Israeli population, may be due to dental trauma or to hereditary factors. Delay in tooth eruption may also be a factor, as evident by the increased prevalence of missing canines among younger than older offspring of native Israelis. We have no reason to expect that missing canines due to the above reasons would be different in the Ethiopian population. On the other hand, the similar rates of missing canines for younger and older offspring of Ethiopian parents suggest that canine bud removal occurred at a young age, under 18 months. The lack of information from the children’s parents regarding the practice of canine bud removal is a limitation of this study.

Dental defects presented in more than 30% of the Ethiopian children in both age groups compared to 4% and 6% of the younger and older offspring of native Israelis, respectively. The latter concurs with a rate of 6% enamel hypoplasia reported in a sample of children in Florida [15]. Enamel defects in teeth adjacent to missing primary canines, as observed in the current study, are a later consequence of canine bud removal, and increase the risk for caries, sensitivity and tooth breakdown [16].

In the current study both hypoplasia and opacities are included in the definition of dental defects. Canine bud removal is an environmental cause of enamel hypoplasia. Enamel hypoplasia is clinically important because it can result in increased susceptibility to caries, wearing of teeth, and tooth sensitivity, as well as impair esthetics. The presence of enamel hypoplasia may inform about a child’s early environment and may be predictive of similar disturbances in permanent dentition [12]. Enamel opacities represent a mild disruption of enamel formation compared to enamel hypoplasias [17].

Presumed alleviation of fever and diarrhea are at the basis for the common practice of tooth bud extraction in young children, common in African countries [1-5,7-11]. In a study of 1050 children and their parents, Kikuilu et al. reported that the major symptoms that led parents to turn to a traditional healer were: persistent fever, diarrhea, vomiting, weight loss, failure to suckle, and crying with unknown cause [10]. The deciduous tooth follicles most often removed are lower canines; the removal is always bilateral [6,8,10].

Evidence of the persistence of canine bud removal in a modern western society highlights the importance to healthcare providers of understanding cultural beliefs and barriers when serving a growing population of immigrant patients [18]. The stress of migration can lead to depression, sadness, reduced self-confidence, personal and family crises, low utilization of health services, and unfavorable health behavior [19]. Hodes found the phenomenon of traditional healers to be far less common in North America than in Israel [20]. He proposed that in North America the Ethiopian population is younger and less cohesive. Though they are educated and integrated into society, they still hold many traditional beliefs or revert to them in times of illness. However, among the very large ethnic group that resides in Israel there seem to be many traditional healers, and the continuation of many traditional practices [20].

Nowadays, in the era of globalization, many people move from one part of the world to another. Nevertheless, immigrants are interested in cultural socialization, including teaching their children about their ethnic customs and traditions. While it is important for immigrant children to keep their cultural identity, the reliance on healers rather than evidence based medicine and dentistry entails health risks. Culturally sensitive educational interventions on oral health are needed [21] to inform health policy and oral health education.

The practice of canine bud removal is well known in Africa. However, the current study demonstrated its persistence among individuals of African descent who lived 20 and more years in a developed country, namely Israel. Documentation of this phenomenon highlights the need to target resources to educate this population using workshops, debates, fliers and other educational tools, as described by Jamieson [22].

## Conclusions

The prevalence of missing primary canines and dental defects was greater among offspring of parents who had emigrated from Ethiopia 15–20 years earlier than among offspring of native Israeli parents living in the same neighborhoods. These findings should prompt public authorities to take measures to end this practice. Parental education of the detrimental consequences of canine bud removal is necessary, as part of a comprehensive program in oral health.

## Competing interests

The authors have no conflicts of interest to disclose.

## Authors’ contributions

ED: Dr. D conceptualized and designed the study, participated in the performance of the research, participated in data analysis, drafted the initial manuscript, and approved the final manuscript as submitted. EK: Dr. K conceptualized and designed the study, and approved the final manuscript as submitted. JS: Prof. S designed the data collection instruments, and coordinated and supervised data collection, critically reviewed the manuscript, and approved the final manuscript as submitted. DR: Prof. R conceptualized and designed the study, participated in data analysis, drafted the initial manuscript, critically reviewed the manuscript, and approved the final manuscript as submitted. All authors read and approved the final manuscript.

## Pre-publication history

The pre-publication history for this paper can be accessed here:

http://www.biomedcentral.com/1472-6831/13/34/prepub
